# Draft Genome Sequence of *Enterobacter aerogenes, *a DDE-Degrading and Plant Growth-Promoting Strain Isolated from *Cucurbita pepo*

**DOI:** 10.1128/genomeA.00317-15

**Published:** 2015-04-16

**Authors:** Nele Eevers, Jonathan D. Van Hamme, Eric M. Bottos, Nele Weyens, Jaco Vangronsveld

**Affiliations:** a Centre for Environmental Sciences, Hasselt University, Agoralaan Building D, Diepenbeek, Belgium; b Department of Biological Sciences, Thompson Rivers University, Kamloops, British Columbia, Canada

## Abstract

We report here the draft genome of *Enterobacter aerogenes*, a Gram-negative bacterium of the *Enterobacteriaceae* isolated from *Cucurbita pepo* root tissue. This bacterium shows 2,2-*bis*(*p*-chlorophenyl)-1,1-dichloroethylene (DDE)-degrading potential and plant growth-promoting capacity. An analysis of its 4.5-Mb draft genome will enhance the understanding of DDE degradation pathways and phytoremediation applications for DDE-contaminated soils.

## GENOME ANNOUNCEMENT

DDT [2,2-*bis*(*p*-chlorophenyl-1,1,1-trichloroethane] (1) is a pesticide that has been used in agriculture and gardening since 1943 (2). In soils, DDT degrades to 2,2-*bis*(*p*-chlorophenyl)-1,1-dichloroethylene (DDE). Both DDT and DDE are classified as persistent organic pollutants (3) and are of concern to human and animal health because of their toxicity and hormone-disrupting properties (4). Endophyte-enhanced phytoremediation using *Cucurbita pepo*, a DDE-accumulating plant (5), is being explored for a role in the remediation of DDE-contaminated soils.

A DDE-degrading bacterial strain was isolated from the roots of *C. pepo* plants exposed to 100 mg·liter^-1^ DDE. Identified as *Enterobacter aerogenes* by partial 16S rRNA gene sequencing and phenotypic profiling, the closest related 16S rRNA sequence (87%) was from *E. aerogenes* strain EA1509E (GenBank accession no. FO203355.1) (6).

Genomic DNA was isolated using a DNeasy blood and tissue kit (Qiagen, Venlo, The Netherlands). An Ion Torrent PGM (Life Technologies, Inc., Carlsbad, CA) was used for sequencing. The DNA was digested, and sequencing adapters were ligated using an Ion Xpress Plus fragment library kit (Life Technologies, Inc., Burlington, Ontario, Canada), according to the manufacturer’s instructions. Adapter-ligated DNA was size selected to a target length of 480 bp on a 2% E-Gel SizeSelect agarose gel, and Agencourt AMPure XP beads (Beckman Coulter, Mississauga, Ontario, Canada) were used for the purification steps. An Ion library quantitation kit was used to calculate the library dilution factor prior to amplification and enrichment with an Ion PGM Template OT2 400 kit on an Ion OneTouch 2 system. An Ion Sphere quality control kit was used to quantify the percent enriched Ion Sphere Particles prior to sequencing with an Ion PGM 400 sequencing kit.

In total, 1.67 million reads (mean length, 298 bases) generated 499 Mb of data in Torrent Suite 4.2.1. These were assembled using MIRA 3.9.9 (7) into 69 contigs >500 bp, giving a consensus length of 4,474,344 bp at 51 × coverage (largest contig, 602,746 bp; *N*_50_, 139,424 bp). Mauve (8) was used to order contigs using the genome of *E. aerogenes* EA1509E (GenBank accession no. FO203355.1) (6), and the annotation was completed using the PGAP (NCBI) pipeline (9). The genome of *E. aerogenes* consists of a single circular chromosome (53.8% G+C content), which includes 4,191 coding genes that were arranged into 309 pathways using Pathway Tools (10, 11), 260 pseudogenes, 40 rRNAs (5S, 16S, 23S), 77 tRNAs, and 7 noncoding RNAs (ncRNAs).

This *E. aerogenes* strain showed increased growth when exposed to DDE in comparison to that under control conditions. Analyses of the draft genome showed the presence of dioxygenases and hydrolases that have been associated with DDE degradation (12–15). Genes for plant growth-promoting capacities, 1-aminocyclopropane-1-carboxylate deaminase activity, siderophore production, auxin biosynthesis, and phosphorous solubilization, are present, corroborating the results from phenotypic tests. These characteristics make *E. aerogenes* a promising strain for DDE phytoremediation.

### Nucleotide sequence accession numbers. 

This whole-genome shotgun project has been deposited at DDBJ/EMBL/Genbank under the accession no. JXTQ00000000. The version described in this paper is version JTXQ01000000.
